# Green Electrospun Poly(vinyl alcohol)/Gelatin-Based Nanofibrous Membrane by Incorporating 45S5 Bioglass Nanoparticles and Urea for Wound Dressing Applications: Characterization and In Vitro and In Vivo Evaluations

**DOI:** 10.1021/acsomega.4c01102

**Published:** 2024-05-02

**Authors:** Tülay Merve Temel-Soylu, Ceren Keçeciler-Emir, Taha Rababah, Cem Özel, Sevil Yücel, Yeliz Basaran-Elalmis, Dilan Altan, Ömer Kirgiz, İlke Evrim Seçinti, Ufuk Kaya, Muhammed Enes Altuğ

**Affiliations:** † Faculty of Chemical and Metallurgical Engineering, Department of Bioengineering, Yildiz Technical University, 34220 İstanbul, Türkiye; ‡ Faculty of Rafet Kayis Engineering, Genetic and Bioengineering Department, Alanya Alaaddin Keykubat University, 07425 Antalya, Türkiye; § Nutrition and Food Technology Department, Jordan University of Science and Technology, Irbid 3030, Jordan; ∥ Faculty of Veterinary, Department of Clinical Sciences, Hatay Mustafa Kemal University, 31060 Hatay, Türkiye; ⊥ Faculty of Medicine, Department of Pathology, Hatay Mustafa Kemal University, 31060 Hatay, Türkiye; # Faculty of Veterinary, Department of Biostatistics, Hatay Mustafa Kemal University, 31060 Hatay, Türkiye

## Abstract

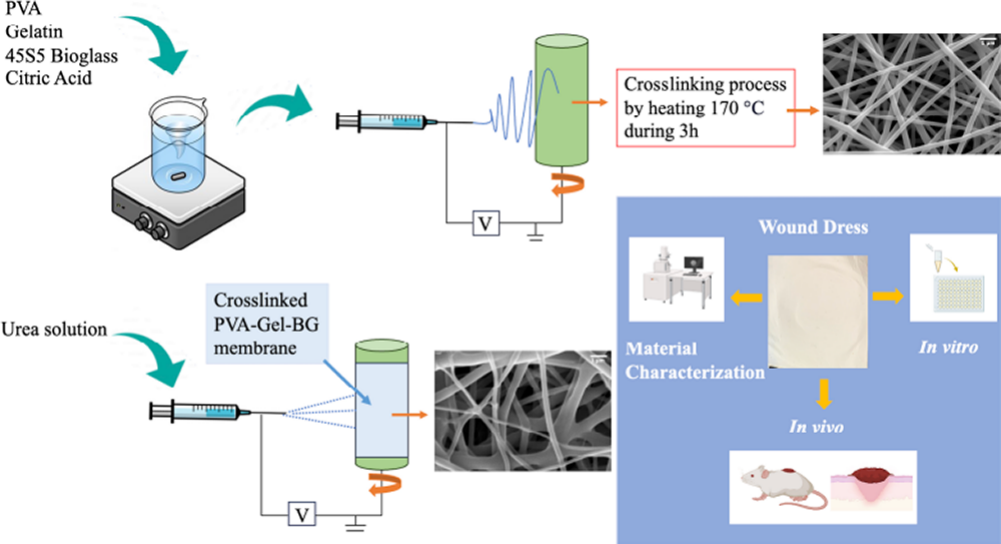

This study reports the fabrication and characterization of poly(vinyl alcohol) (PVA) and gelatin (Gel)-based nanofiber membranes cross-linked with citric acid (CA) by a green electrospinning method in which nano 45S5 bioglass (BG) and urea were incorporated. Various combinations of PVA, gelatin, and BG were prepared, and nanofiber membranes with average fiber diameters between 238 and 595 nm were fabricated. Morphological, chemical, and mechanical properties, porosity, swelling, water retention, and water vapor transmission rate of the fabricated membranes were evaluated. PVA:Gel (90:10), 15% CA, and 3% BG were determined as the optimum blend for nanofiber membrane fabrication via electrospinning. The membrane obtained using this blend was further functionalized with 10% w/w polymer urea coating by the electrospray method following the cross-linking. In vitro biocompatibility tests revealed that the fabricated membranes were all biocompatible except for the one that functionalized with urea. In vivo macroscopic and histopathological analysis results of PVA/Gel/BG and PVA/Gel/BG/Urea treated wounds indicated increased collagenization and vascularization and had an anti-inflammatory effect. Furthermore, careful examination of the in vivo macroscopic results of the PVA/Gel/BG/Urea membrane indicated its potential to decrease uneven scar formation. In conclusion, developed PVA/Gel/BG and PVA/Gel/BG/Urea electrospun membranes with multifunctional and biomimetic features may have the potential to be used as beneficial wound dressings.

## 1 Introduction

Globally, acute and chronic wounds substantially influence an individual’s well-being and entail considerable financial burdens for healthcare systems.^1^ The worldwide market for advanced wound care, including wound dressings, is expected to grow from £14.8 billion in 2019 to £18.6 billion in 2024.^1^ Furthermore, the treatment of skin scars is an additional expense associated with wound healing, a $12 billion annual market.^2^

Wound healing is a crucial and highly organized process that allows skin to maintain its function as a protective barrier.^3^ Natural wound healing occurs in three main phases: inflammation, growth, and remodeling. Several factors, such as pressure, temperature, oxygen levels, and wound’s moisture, play a pivotal role in wound healing.^3,4^ Acute wounds typically follow the normal healing process and close within 8 to 12 weeks.^5^ In contrast, chronic wounds take more than 3 months to heal due to complications like infection, diabetes, and peripheral vascular diseases.^6,7^ Additionally, scar formation is an additional challenge associated with wound healing, which can have significant functional and aesthetic consequences. Both hypertrophic and normal scars are hard to completely prevent and difficult to treat.^2^

Wound dressings have been developed to protect the wound from infection and accelerate the wound healing process.^8,9^ Traditional dressings are widely utilized due to their low cost, but they have some limitations including their inability to maintain wound moisture, adhesion to granulation tissue, and ineffectiveness in preventing the ingress of microorganisms.^9,10^ Because of the limitations of traditional wound dress, researchers focus on development modern wound dressings in order to eliminate the multifaceted obstacles in the wound healing process.^9^^–^^11^ Modern dressings have considerable advantages such as biocompatible, biodegradable, exudate management, gas permeability, moisture regulation, microbial inhibition, and mechanical stability.^8^^–^^10,12^ Modern dressings come in various forms, including hydrogels, hydrocolloids, alginates, foams, films, and membranes.^9,11,12^

Since electrospinning is a simple, fast, and efficient method, it is widely used for the production of nanofiber dressings.^13^ Electrospun fiber mats have notable properties including high surface area, high porosity, the ability to functionalize fiber surfaces for the modulation of physical and chemical attributes, enhanced capacity for exudate absorption, augmented permeability to water and oxygen, as well as the presence of small pore diameters related to effectively mitigating the infiltration of exogenous.^14,15^ Moreover, the fibrous architecture generated through the process of electrospinning bears a resemblance to the natural extracellular matrix (ECM) and promotes cell attachment and proliferation.^16^ These advantages make the electrospinning method to be at the forefront of biomaterial production in recent years.^17^ The electrospinning method is suitable for use in the application of both natural and synthetic polymers. Through the fabrication of electrospun composite matrices, it becomes feasible to integrate the robust mechanical characteristics and reduced degradation rates of synthetic biopolymers with the inherent bioactive properties of natural polymers.^16,17^

Poly(vinyl alcohol) (PVA) is a semicrystalline water-soluble synthetic polymer which has notable characteristics such as suitable chemical and thermal stability, biocompatible, biodegradable, and biomechanical.^13,18−20^ Especially, it stands out in the production of wound dressings due to its nanofiber material production capacity by electrospinning.^13^ PVA can be easily cross-linked by physical (freeze–thawing, UV exposure, and heat treatment) or chemical (some alde-hydes such as glutaraldehyde, formaldehyde, or glyoxal and poly(carboxylic acid)s such as 1,2,3,4-butane-tetracarboxylic acid, citric acid) methods.^21,22^ Citric acid is a natural polysaccharide containing one hydroxyl and three carboxyl groups; it can be utilized as natural cross-linking agent which allows green electrospinning, and it cross-links PVA by forming ester bonds between its carboxyl groups and the hydroxyl groups of PVA at high temperature.^23,24^

Gelatin (Gel) is a biocompatible and biodegradable natural polymer derived from collagen.^25^ Moreover, gelatin has peptide sequences that bind to integrin receptors in cells, which helps it play an essential role in promoting cell adhesion in wound dressing applications.^25^ Despite its cost-effectiveness, its suitability for use in biomedical applications is limited by certain drawbacks, such as a tendency for degradation and reduced mechanical strength. These challenges can be overcome by combining gelatin with polymers like poly(vinyl alcohol) (PVA).^26,27^

The utilization of bioglasses for bone regeneration has been well-documented, owing to their properties in stimulating osteogenic cells.^28^ Recent studies have revealed that 45S5 bioglass (by weight 45% SiO_2_, 24.5% CaO, 24.5% Na_2_O, and 6.0% P_2_O_5_) can interact with soft tissues and is effective in accelerated wound healing by promoting angiogenesis and increasing the expression of vascular endothelial growth factor (VEGF).^29^^–^^31^ In addition, it has been reported that 45S5 bioglass nanoparticles accelerate blood coagulation and increase the surface roughness, wettability, and overall biocompatibility of wound dressing materials.^32^

Urea is required for moisturizing, keratolytic action, antimicrobial defense, regulation of epidermal proliferation, and barrier function of the skin. Because urea is highly soluble in water, it is frequently used in dermatology. The effects of urea at different concentrations are as follows: moisturizing and optimizing the barrier function of the skin at low concentrations (2–10%), moisturizing and acting as a keratolytic agent at medium concentrations (10–30%), and acting as a keratolytic agent and removal of necrotic tissue at high concentrations (≥30%).^33^

The aim of this study was to fabricate a biocompatible PVA/Gel based wound dressing material with added functionalities that would be provided by the inclusion of sol–gel 45S5 nanobioglass and urea. One of the goals of this study was to discover the influence of bioglass incorporated PVA/Gel membrane on wound healing as well as the synergistic effect of urea and bioglass by evaluating the effect of the PVA/Gel membrane containing both urea and bioglass on wound healing. In this context, a widely preferred electrospinning technique was selected to fabricate PVA/Gel materials due to the similarity of nanofiber structures provided by this technique to ECM. Electrospun membranes were characterized by means of morphology, fiber diameter, chemical stability, mechanical properties, porosity, and swelling degree, which were evaluated for the selection of the most suitable membrane for wound dressing applications. The membrane selected was modified with urea via the electrospraying technique in order to improve the skin’s barrier function and moisturize the wound environment. In vitro biocompatibility of the membranes was evaluated in both the presence and absence of urea. The wound dressing characteristics of the membranes were systematically investigated through the utilization of an in vivo animal experimental framework, involving the assessment of wound closure rate and a thorough histopathological analysis. A promising potential for use as an effective wound dressing material was shown by the developed PVA/Gel/BG and PVA/Gel/BG/Urea nanofibrous membranes.

## 2 Materials and Methods

### 2.1 Materials and Reagents

Poly(vinyl alcohol) (PVA, molecular weight 89,000–98,000, 99%+ hydrolyzed) and urea (≥99.0%) were purchased from Sigma-Aldrich Chemical Co. Gelatin was purchased from AppliChem (Darmstadt, Germany). Citric acid was purchased from Merck KGaA (Darmstadt, Germany). 45S5 Bioglass (99.07 nm average particle size, 2.2912 m^2^/g BET surface area) was produced in nanosize with the sol–gel method by Keçeciler-Emir et al. at Yildiz Technical University (Istanbul, Turkey).^34^

Wistar rats (200–300 g, 6–8 weeks old) were purchased from the Hatay Mustafa Kemal University Experimental Research and Application Center. The study approval was obtained from the Local Ethics Board of Animal Experiments of Hatay Mustafa Kemal University (Decision No. 2021/01-15). Experiments were performed in accordance with the Turkish Code of the Welfare and Protection of Animals Used for Experimental and Other Scientific Purposes and Directive 2010/63/EU on the protection of animals used for scientific purposes.

### 2.2 Electrospinning of PVA/Gelatin with Bioglass

The electrospinning solutions were prepared in pure deionized water. Poly(vinyl alcohol) (PVA) was dissolved 15% (w/w) at 80 °C for 4 h in a magnetic stirrer. Gelatin was dissolved 12.5% (w/w) at ambient temperature in a magnetic stirrer until it was completely dissolved. PVA and gelatin solutions were combined into three different volumes of 90:10, 85:15, and 80:20. Bioglass was added to mixtures at various concentrations (1, 2, and 3 wt %). Citric acid used as a cross-linking agent was added to mixtures at various concentrations (10, 15, and 20 wt %).^23,35^ The mixtures were stirred (500 rpm) for 5 min, and degassing treatment and homogenization were carried out by holding it in an ultrasonic water bath at 50 °C for 15 min. The amounts of bioglass, citric acid, and urea were calculated in relation to the total weight of the polymers in the mixtures.

The mixtures were placed in the syringe pump with a 10 mL plastic syringe. The distance between the pump and the collector is 10 cm, and the plastic syringe is connected at a voltage of 18 kV. The flow rate of the solution is 500 μL under ambient conditions and humidity varies between 22 and 37%. The drum was used as a collector, and the drum rotation speed was set to 300 rpm. Since the cross-linking with citric acid is at the desired level at 170 °C, it was determined for the cross-linking process.^36^ The fibers were cross-linked in the oven for 3 h. Table 1 presents the contents of the developed nanofiber membranes.

**Table 1:** Developed Nanofiber Membranes via Electrospinning and Electrospraying with Different Contents

membranes	PVA:gel	bioglass (wt %)	citric acid (wt %)	urea (wt %)
M1	80:20		15	
M2	80:20	1	10	
M3	80:20	1	15	
M4	80:20	1	20	
M5	85:15		15	
M6	85:15	1	15	
M7	85:15	2	15	
M8	90:10		15	
M9	90:10	1	15	
M10	90:10	2	15	
M11	90:10	3	15	
M12	90:10	3	15	10

### 2.3 Production of Urea-Containing PVA/Gelatin/BG Wound Dressing

The membrane containing the combination PVA:gelatin 90:10, bioglass 3 wt % and citric acid 15 wt % was wrapped in a drum after cross-linking. 10% urea was preferred in the study since 10% urea concentration was reported to have the greatest effect on improving skin hydration.^37^ 10% (w/w polymer) urea by weight of polymer in the membrane was dissolved in pure deionized water. The urea solution was placed in a 12 kV voltage-connected syringe and electrosprayed onto the membrane surface. The distance was 10 cm from the collector to the pump, the drum rotor speed was 200 rpm, and the flow rate was 3500 μL/h. The obtained membrane was dried in a vacuum oven at 50 °C for 2 h.

### 2.4 Material Characterization

#### 2.4.1 Morphological Assessment of Fibers

The morphology of the produced fibers was determined by scanning electron microscopy (SEM; Carl Zeiss, EVO Ls 10 T, Germany). Prior to the SEM analysis, gold was sputtered on the surfaces of all samples under a vacuum with the sputter coater device (Emitech, Emitech K550X, UK). Java’s ImageJ software (version 1.53t) was used to calculate the average diameter of the fiber, and 60 fiber diameters were measured for each sample. The BG and urea contents in the membrane were determined by energy-dispersive X-ray spectroscopy (EDS; Carl Zeiss, SmartEDX) by point analysis with three different points.

#### 2.4.2 Infrared Spectroscopy

The chemical bonds and functional groups of the fibers were recorded on a Fourier transform infrared spectrometer (FTIR; Shimadzu Corporation, IRPrestige-21, Japan). IR spectra in transmission mode were obtained in the spectral regions of 650–4000 cm^–1^, and IR spectra were obtained by collecting 15 scans, each spectrum of the samples, with a resolution of 2 cm^–1^.

#### 2.4.3 Mechanical Properties

The mechanical properties of the membranes were performed in tension mode at room temperature using dynamic mechanical analysis (DMA; Devotrans- GPUG/R, Turkey) in accordance with the ASTM D882–10 protocol with minor modifications [ASTM (2010), Standard test method for tensile properties of thin plastic sheeting (ASTM D882–10 2010), Annual Book of ASTM Standards, American Society for Testing and Materials, Philadelphia, PA]. Membranes with dimensions of 1 cm in width and 3 cm in length were utilized. Prior to measurements, the thickness of the films was determined by averaging measurements taken randomly by using a caliper. The tensile test was performed at a test speed of 5 mm/min and a preload speed of 0.1 mm/min.^38^

#### 2.4.4 Porosity

The porosity of the membranes was calculated using the ethanol displacement technique. First, the membranes were prepared by cutting (1 cm × 2 cm). The thicknesses of the membranes were measured using a digital caliper (Insize SL-1108-200), and the volumes of the membranes were calculated. The dry weights (*W*_0_) of the membranes were weighed and then soaked in ethanol for 1 h, and the saturation weights (*W*_1_) were recorded. The percentage of porosity was calculated using the following formula:^39^
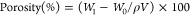
1Here, *ρ* (g/cm^3^) indicates the density of ethanol and *V* (cm^3^) represents the volume of the membrane.

#### 2.4.5 Swelling Degree

The degree of swelling of the membranes was determined by using liquid absorption capacity. First, the membranes were cut (1 cm × 1 cm). They were dried in an oven at 105 °C for 2 h and their dry weights (*W*_d_) were recorded. The membranes were kept in distilled water at ambient conditions (25 °C) for 24 h, and then the excess surface water was removed using filter paper and weighed (*W*_s_). The percentage of swelling was calculated using the following formula:^40^
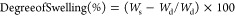
2

#### 2.4.6 Water Vapor Transmission Rate

The ASTM E-96-00 desiccant method, with slight modifications, was employed to perform measurements of water vapor transmission (WVT) and water vapor permeability (WVP) for the wound dressing fiber membranes.^40^ Blue silica beads were added to the membranes as a desiccant agent at a rate of approximately 400 mg into 1000 μL pipet tips with the ends of the tips closed, and the membranes were sealed in between with 200 μL pipet tips with cut ends. The total initial weight of the beads was measured and recorded. The experimental setup was subsequently filled with distilled water and transferred to a desiccator maintained at 37 °C and 90% relative humidity (RH) for 24 h. WVT and WVP were determined based on the weight change of the beads at 24 h using the following equation:^41^
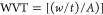
3where *w*/*t* is the weight increase with time and *A* is the surface area of the membrane (m^2^).
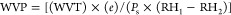
4where *e* is the average thickness of the membrane (m), *P*_s_ is the water vapor saturation pressure at the measuring temperature, RH_1_ is the relative humidity in the desiccator, and RH_2_ is the relative humidity in the tips.

### 2.5 In Vitro Characterization

#### 2.5.1 In Vitro Cell Viability Test

The cell viability assay of the membranes was performed on the L929 fibroblast cell line based on the MTT (3-[4, 5-dimethylthiazol-2-yl]-2, 5-diphenyl tetrazolium bromide) technique according to the ISO 10993-5:2009 procedure. Before the test, the membranes were sterilized under UV light for 30 min in both directions. The ISO 10993-12:2012 standard was taken as a reference for the extraction of the membranes. Therefore, all sterile membranes were weighed at 0.1 g. The membranes were extracted in Dulbecco’s modified Eagle’s medium (DMEM) at 37 °C for 24 h, and the extracts of the membranes were used after sterilization with using sterile 0.22 μL syringe filter for cytotoxicity testing. L929 fibroblast cells (1 × 10^5^ cell/mL) were seeded into a 96-well plate and incubated for 24 h. 50 μL of MTT dye were added to each well of the plate, after 2 h of incubation the colorimetric density (absorbance value) was measured at 570 nm (reference wavelength 650 nm) using a Microplate Reader. The positive control was 1% phenol solution, whereas the negative control was DMEM. Results were calculated assuming the negative control as 100% viable. The percentage of cell viability was calculated using the following equation:^42^
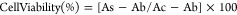
5

which includes As, absorbance of cell-extract interaction; Ac, absorbance of cell with no extract interaction; Ab, absorbance of blank dimethyl sulfoxide (DMSO).

### 2.6 In Vivo Wound Healing Assay

#### 2.6.1 Animal

The in vivo experiment was carried out using forty-eight healthy adult male Wistar rats (200–300 g, 6–8 weeks old) in Hatay Mustafa Kemal University Experimental Research and Application Center. One week prior to the study, the animals were taken to the study place to undergo routine health checks, and time for adaptation was provided. Rats were kept at a controlled temperature (22° ± 2 °C) and 12 h photoperiod throughout the study.

#### 2.6.2 In Vivo Experimental Design and Wound Area Measurement of Developed Wound Dressings

Following the induction of general anesthesia (ketamine HCl 50 mg/kg and xylazine HCl 10 mg/kg, ip), the back hair of the rats was shaved, and the area was sterilized with povidone iodine. A 1 × 1 cm (1 cm^2^) full-thickness excisional wound was created on the dorsal side of all rats. The animals were randomly divided into four experimental groups (*n* = 12 each);

*The Sham group (Group S):* Wounds were not covered with any dressing and were kept as negative control.

*M8 group (Group M8):* Wounds were covered with the membrane containing PVA:Gel (90:10)%15CA (M8 membrane).

*M11 group (Group M11):* Wounds were covered with the membrane containing PVA:Gel (90:10)%15CA%3BG (M11 membrane).

*M12 group (Group M12):* Wounds were covered with the membrane containing PVA:Gel (90:10)%15CA%3BG-Urea (M12 membrane).

All treatments were applied once to cover the wound area. All groups were divided into 7 and 14 day subgroups in order to compare the recovery degrees on the seventh day and the 14th day and placed in individual cages (*n* = 6 each). On the seventh and 14th day, the rats were deeply anesthetized (xylazine HCl 10 mg/kg and ketamine HCl 100 mg/kg, ip) and euthanized. The dorsal line wound area was excised with a surgical blade and scissors. Samples from each animal were removed for histopathological and immunohistochemical analyses in 10% formalin solution. The wound areas of all animals were photographed individually by a digital camera at 0, 1, 3, 5, 7, 10, and 14 days after wound creation. The wound surface area (cm^2^) was measured using ImageJx2 software.

#### 2.6.3 Histopathological and Immunohistochemical Analysis of Wound Sites

Biopsies were taken on days 7 and 14 for microscopic evaluation. After 24 h of fixation in 10% neutral buffered formalin, the skin and subcutaneous tissues were excised for standard histological processing, centered on the incision line. Samples were fixed in 10% neutral buffered formalin, dehydrated in graded alcohols, and then embedded in paraffin. Sections of 4 μm thickness were made and stained with hematoxylin-eosin (HE) and Masson’s trichrome (MT) using routine histological protocols. Re-epithelialization and inflammation were evaluated in HE stained sections; in order to evaluate the re-epithelialization, the epithelial thickness on the wound area was measured from the basal layer to the uppermost layer thickness from 3 different areas, and the average was calculated using an Olympus DP2BSW software. For the evaluation of inflammation, the work of Atıcı et al. has been modified.^43^ After the wound area was scanned at ×100 magnification (BBA), the area with the most intense inflammation was selected and inflammatory cell count was performed at ×200 BBA. Neutrophil leukocytes (PMNL) to assess acute inflammation, lymphocytes, macrophages, and plasma cells to assess chronic inflammation were counted and scored according to their sum:^43^

Score 0:0–4 inflammatory cells/200 BBA
Score 1:5–20 inflammatory cells/200 BBA
Score 2:21–80 inflammatory cells/200 BBA
Score 3: >80 inflammatory cells/200 BBA

The ratio of collagen fibers stained blue with Masson trichrome to the tissue covering ×200 BBA was calculated by using Java’s ImageJ software (version 1.53t) and scored as follows.^43^

Score 0: none
Score 1: <10% collagenization
Score 2: 10–49% collagenization
Score 3: ≥50% collagenization

Neovascularization was evaluated by immunohistochemical study; tissue sections were deparaffinized in xylene and then rehydrated in graduated concentrations of ethyl alcohol (100%, 96%, 80%, 70%, and water). Anti-CD 31(DAKO, Monoclonal Mouse Anti-Human, Clone JC70A, ready-to-use, Danmark) was microwaved in citrate buffer to induce antigen recovery for antibody (1/50 dilution, Dako). Endogenous peroxidase activity was blocked by incubating the slides in peroxidase blocking solution for 5 min. The EnVision Detection Kit (Env FLEX, High pH, DAKO) was used as the stain detection system. Sections were counterstained with hematoxylin, dehydrated with ethanol, and permanently covered with a coverslip. Microvascular density was evaluated in anti-CD31 monoclonal antibody-applied preparations. Microvascular density was measured as the number of new microvessels per 200× magnification optical field. Three random areas of high vascular density at 100× magnification were selected, and microvessels were counted at ×200 magnification. The final microvessel density score was calculated as the mean number of vessels in these three areas. Vessels containing muscle layers were not included in the microvessel count.

All samples in each group were systematically evaluated using an Olympus BX51 (Olympus corp., Tokyo, Japan) light microscope, on an Olympus DP72 microscope digital camera system, and Olympus DP2BSW software for inflammation, collagenization, epithelialization, and neovascularization.

### 2.7 Statistical Analysis

Before performing the statistical analysis, data were examined for normality as parametric test assumptions. Descriptive statistics for each variable were calculated and presented as the “mean ± standard error of mean”. Statistical analysis for porosity, swelling test, water vapor transmission rate, cell viability (%), wound closure (%), epithelial thickness, and the number of newly formed wound vessels was performed using one-way analysis of variance (ANOVA) followed by Tukey’s multiple comparison test. Statistical analysis for inflammation and collagenization was performed using the Kruskal–Wallis test followed by Dunn’s test for multiple comparisons. Stata version 16.1 (StataCorp, College Station, TX, USA) and GraphPad Prism statistical software (GraphPad Software Inc., USA) were used for analysis of the data.

## 3 Results and Discussion

### 3.1 Morphology and Size of Fibers

**Figure 1:**
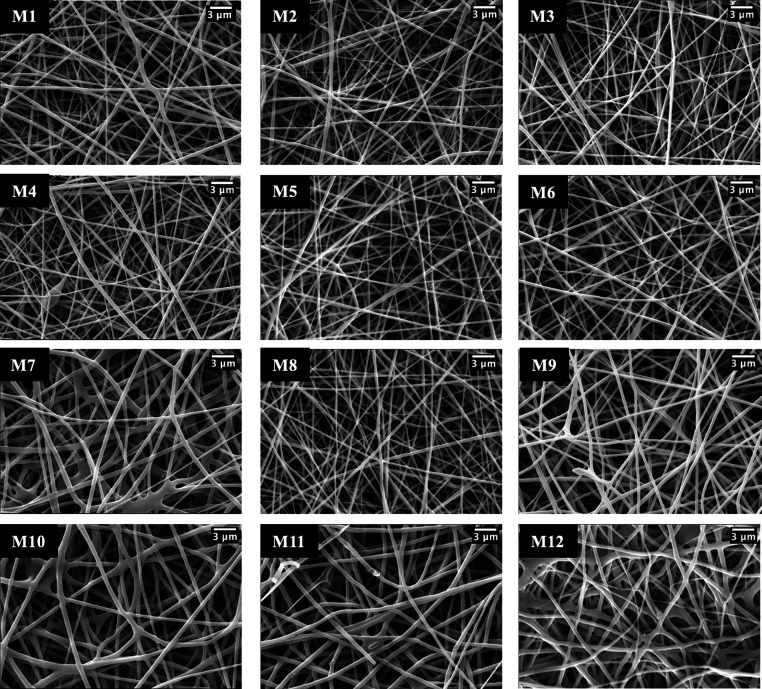
SEM images of PVA/gelatin membranes with different ratios and contents at ×10k magnification.

The morphology of the prepared membranes with different PVA gelatin ratios (90:10, 85:15, 80:20) and different BG (0–3% w/w) and %10 w/w urea content was examined by scanning electron microscopy (SEM), and the results are shown in Figure 1. All nanofibers have generally uniform, smooth surfaces and are bead-free. Table 2 displays the mean diameters of the nanofibers, which were calculated by using ImageJ on the SEM image.

**Table 2:** Fiber Diameters of PVA/Gelatin Membranes with Different Ratios and Contents

membranes	average fiber diameter (nm)
M1	238 ± 60
M2	260 ± 82
M3	248 ± 54
M4	208 ± 74
M5	265 ± 67
M6	322 ± 71
M7	472 ± 158
M8	287 ± 69
M9	393 ± 50
M10	489 ± 99
M11	547 ± 68
M12	595 ± 158

It was known that with the addition of citric acid directly to the blend as a cross-linker, the pH of the blend decreased and the electrical conductivity increased since the ionization of citric acid and the viscosity of the blend did not change.^23,35^ In this context, various ratios of citric acid from 10 to 20% (w/w polymers) have been tested. It was observed that fiber thicknesses decreased when only the amount of cross-linker was increased from 10 to 20% (w/w polymers) by keeping the bioglass ratio (1% w/w) and polymer composition (PVA:Gel, 80:20) constant. The membrane containing 15% (w/w polymer) citric acid has a smooth and uniform (depending on standard deviation) fiber appearance compared to those of the others. It is stated in the literature that increasing the volumetric ratio of PVA leads to a decrease in the conductivity and the viscosity of the blend increases, the surface charge density and the repulsive force of the jet decrease, and as a consequence, larger fiber diameters are obtained.^44^ Accordingly, it was determined that average fiber diameters of PVA:Gel (80:20) %15 CA (M1), PVA:Gel (85:15) %15 CA (M5), and PVA:Gel (90:10) %15 CA (M8) (only the various combination of PVA:Gel nanofibers) increase as 238, 265, and 287 nm, respectively.

The effect of BG concentration in different compositions of PVA:Gel nanofibers (90:10, 85:15, 80:20) was examined on the thickness of the fiber diameters, and it was observed that the mean fiber diameters and standard deviations increased as the bioglass ratios increased in all polymer ratios. It has been reported by Liverani et al. that the maximum and minimum fiber diameter ranges widen due to the increase in conductivity of the polymeric solution containing BG particles, and accordingly, the standard deviation increases.^45^ In particular, it was observed that in the PVA:Gel (85:15) combination, the fibers became thicker, and some fibers joined each other. Also, a significant increase in mean fiber diameter and standard deviation of PVA:Gel (85:15) %15CA%2BG (M7) was determined. In comparison to these data, the fibers containing 90:10 PVA:Gel seem to be more consistent and smooth. It has been noticed that the urea incorporated M12 fibers produced via the electrospray method have a thicker structure compared to that of the M11 fibers without urea additives. This is thought to be due to the swelling of the fibers by the absorption of water during electrospray.

The presence of BG particles and urea in the membranes was identified via SEM/EDS with data taken from 3 different points. It is verified by Table S1 and Figure S3 that when the amount of 45S5 BG particles in the polymer blend is increased from 1 to 3% (w/w polymer), the amount of 45S5 BG also increases in the membrane. The existence of urea in the M12 membrane was confirmed by increasing the amount of nitrogen in the M12 membrane compared to that in M11. According to this result, it can be concluded that the M12 membrane was produced by successfully coating the surface of the M11 membrane with urea by using the electrospray technique.

### 3.2 FTIR Analysis

FTIR analysis was used to investigate the chemical bonds and specific groups of the produced membranes. The various peaks of PVA, such as the regular stretching vibration peaks of the O–H group from the intra- and intermolecular hydrogen bonds, were visible at wavelengths between 3000 and 3600 cm^–1^, indicating a wide and intense peak. The C–H stretching vibrations of −CH_2_ are responsible for the peaks that appear in the wavelength range of 2930 cm^–1^. The peak at 1485 and 1342 cm^–1^ indicates the scissoring −CH_2_ group and vibrational bending −OH group. The peaks appeared at 1085, 1024,and 845 cm^–1^ are assigned to C–O stretching, C–C stretching, and C–O–C asymmetric stretching, respectively. The various peaks of gelatin indicate the following: the peak at 2932 cm^–1^ indicates the stretching of the −N–H group of the secondary amides in the gelatin structure, and 1657 cm^–1^ wavelength indicates C=O stretching and hydrogen bonding combined with COO- stretching, while the peaks at 1544 and 1236 cm^–1^ indicate N–H and C–N stretching of the amide II group. The peak at 1530 cm^–1^ shows amide I and C–N stretching groups. The peak at 1237.6 cm^–1^ indicates −N–H bending and the amide-III^21,46−49^ The functional chemical groups of the gel and PVA are generally overlapped.

**Figure 2:**
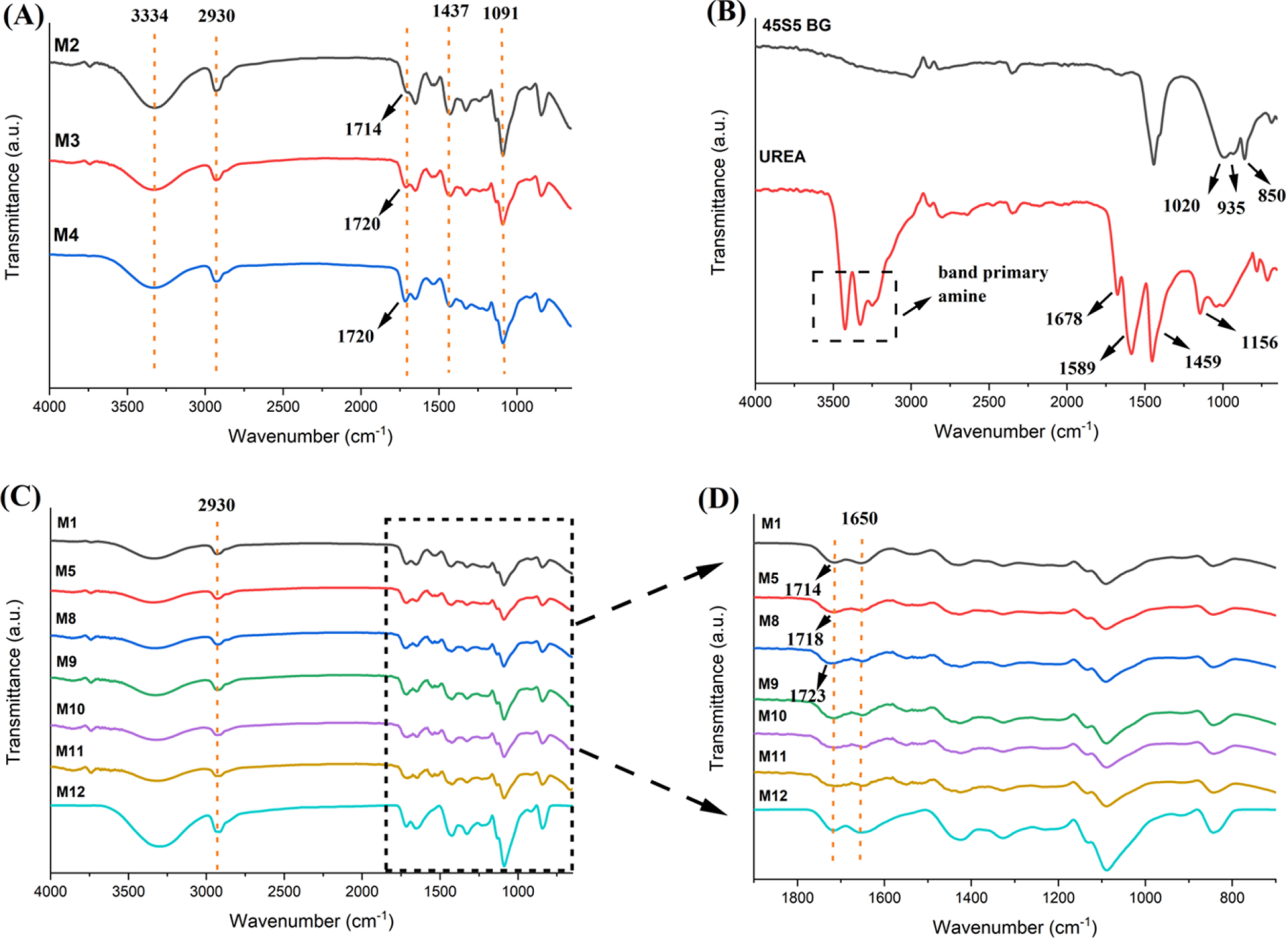
FTIR spectra of (A) various citric acid amount comparison (between samples M2, M3, and M4) and (B) BG and urea, (C) PVA:Gel combination, BG and urea additives comparison (between samples M1, M5, M8, M9, M10, M11, and M12) in the range of 4000–650 cm^–1^, and (D) PVA:Gel combination, BG and urea additives comparison (between samples M1, M5, M8, M9, M10, M11, and M12) in the range of 4000–750 cm^–1^.

The cross-linking occurs because of the esterification reaction between the hydroxyl groups of PVA and the carboxylic groups of citric acid. In Figure 2A, the peaks at 1714 and 1720 cm^–1^ indicate C=O due to the formation of the ester carbonyl group.^23,35^ In Figure 2A, with the increase of the amount of citric acid, it was observed that the peak shifted from 1714 (M2) to 1720 cm^–1^ (M3 and M4) (C=O due to the formation of an ester carbonyl group). In Figure 2D, with the increase of the amount of PVA (M1, M5, and M8), it was observed that the peak shifted from 1715, 1718 to 1723 cm^–1^ (C=O due to the formation of ester carbonyl group), respectively. The peak observed at 3334 cm^–1^ signifies O–H stretching, the peak at 2941 cm^–1^ corresponds to C–H stretching within alkyl groups, the peak at 1437 cm^–1^ denotes CH_2_ bending, and the peak at 1091 cm^–1^ indicates C–O stretching.^23^ In Figure 2A, it has been observed that when the amount of citric acid is increased from 10 to 15% w/w polymer, the intensity of the O–H peak at a wavelength of 3334 cm^–1^ decreases. This observation is associated with the cross-linking.^50^ When the citric acid ratio is 20% (w/w) polymer, it shows the same characteristic as in the 15% (w/w) polymer citric acid ratio. To minimize the nonesterified carboxyl groups of citric acid to avoid high acidity, it was agreed that 15% w/w polymer citric acid ratio was sufficient for the cross-linking of the fibers.

Characteristic peaks of the 45S5 BG are given in Figure 2B. In the SiO_4_ tetrahedron structure, the peak observed at 850 cm^–1^ wavelength displays two unbridged oxygen-containing Si–OH groups, while the peak at 1020 cm^–1^ wavelength displays Si–O–Si groups. The peaks at 935 cm^–1^ that show the presence of crystallization in the sol–gel BGs is caused by the stretch bond between Si and the nonbridging oxygen spectrum.^34,51^ Since the characteristic bands of the bioglass overlap with the bands of the polymers and the amount of bioglass is much less than the polymer, it cannot be detected in the spectra.^52^ The stretching frequency of C=O, C–N, and C–O appeared at 1678, 1459, 1156 cm^–1^, respectively, according to the urea analysis (Figure 2B).^37,53^ The stretching and deformation of the N–H bond was observed at 3427 and 1589 cm^–1^, respectively.^37^ Some characteristic peaks are seen in Figure 2C of the FTIR spectra of PVA/Gel based urea-containing membranes; N–H/O–H overlapping band appears in the range of 3600–3200 cm^–1^. In addition, the urea-containing membrane’s FTIR spectrum was found to be more intense at 2930 cm^–1^ wavelength due to C–H tensile vibrations than the other membranes. Moreover, a wide peak at a wavelength of 1650 cm^–1^ appears in membranes containing urea. In this region, the amide absorption peak is located. The width of the peak shows that the C=O absorption band overlaps with the amide band.^54^

### 3.3 Mechanical Properties

**Figure 3:**
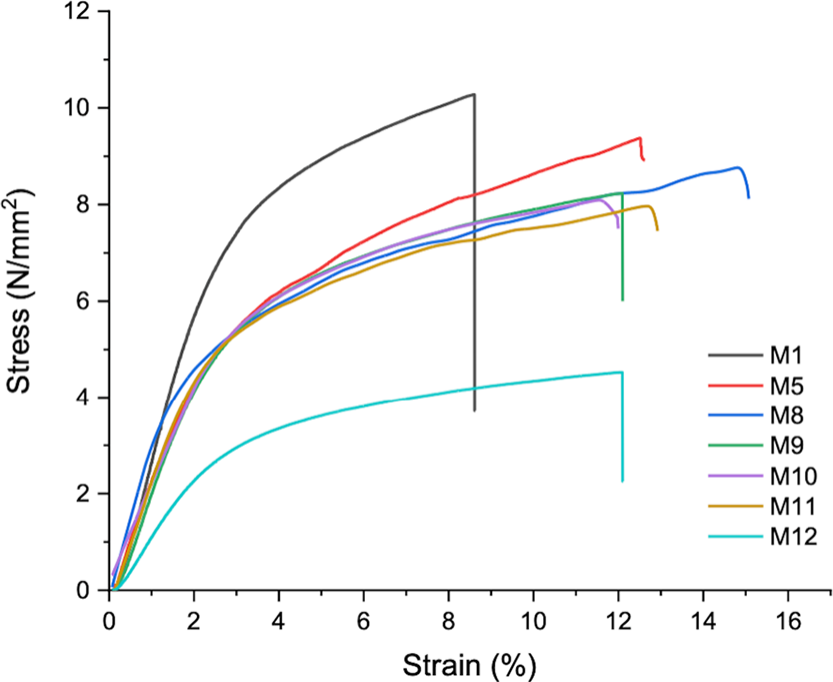
Tensile stress–strain curves of various membranes.

The tensile test serves as a valuable means of assessing the strength of a given sample (Figure 3). For biomaterials intended for use as wound dressings, it is highly desirable that they possess both suitable strength and flexibility.^55^Table 3 shows the tensile strengths, young modulus, and elongation at break values of membranes prepared at different PVA:Gel ratios and bioglass and urea contents. As shown in Table 3, the tensile strength of wound dressing fibers weakens when the PVA content is increased from 80% w/w polymer (M1, 10.28 MPa) to 90% w/w polymer (M8, 8.46 MPa). This is consistent with the results of a similar study performed by Thuy et al. and it has been associated with an increase in fiber diameter (see also Table 2).^27^ The tensile strength of the wound dressing fibers was observed to decrease from 8.46 to 7.76 MPa (M11) upon the addition of bioglass. This can be explained by the fact that the particles (bioglass, etc.) included in the polymer act as rigid inclusions, reducing the strain at break.^56^ It can be said that another reason for the decrease in tensile strength is the increase in fiber diameter of membranes containing bioglass.^27^ In addition, it can be concluded that the urea spraying onto the fibers results in a notable decrease in their tensile strength, which can potentially be attributed to the potential hardening of the fiber following the drying of excess water in the urea solution on the surface of the fiber matrix. In conclusion, it has been observed that all produced membranes are compatible with the mechanical properties of the skin tissue (in the range of 0.1–10 MPa).^30^ The Young’s modulus of membranes with bioglass decreased compared to the control membrane (M8). It can be said that this decrease is due to the increase in the inhomogeneity of fiber diameters of membranes containing bioglass (see also Table 2) as in a previous similar study performed by Liverani et al.^45^ Based on this information, the reason for the decrease in Young’s modulus of the urea-containing membrane (M12) can be attributed to the inhomogeneity of the fiber diameters. When the elongation at break % values of the membranes (M1, M5, and M8) were compared, it was observed that the elongation at break value increased as the PVA amount increased. The reason for this is the abrupt break of gelatin, while PVA breaks gradually due to its elastic behavior.^27^ The elongation at break value did not change significantly with the addition of bioglass and urea to the membrane. These findings suggest that the developed fibers have the potential to serve as ideal materials for wound dressing applications.

**Table 3:** Tensile Strength, Young Modulus, and Elongation at Break Values of the Various Membranes

membrane	tensile strength (MPa)	Young modulus (MPa)	elongation at break (%)
M1	10.28	325.24 ± 0.98	8.61
M5	9.38	245.07 ± 0.84	12.46
M8	8.46	307.46 ± 0.83	13.48
M9	8.22	239.56 ± 0.70	12.04
M10	8.09	199.82 ± 0.14	13.62
M11	7.76	256.50 ± 0.21	13.64
M12	4.52	130.06 ± 0.33	12.04

### 3.4 Porosity

**Figure 4:**
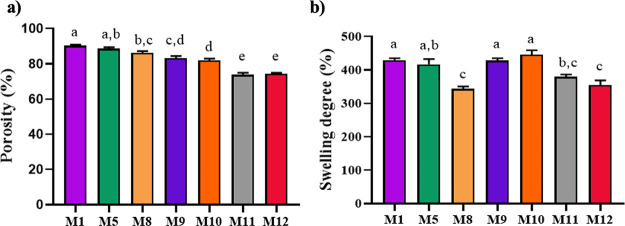
Porosity (a) and swelling degree (b) values of the prepared membranes (data are given as mean ± SEM (*n* = 6)). The different letters on the bar indicate significant differences between values, and the same letters on the bar indicate no difference between values according to Tukey’s multiple comparison test (*p* < 0.05).

The porosity of the wound dressing is an important parameter in wound healing as it supports moisture in the wound area, provides adequate gas and nutrient exchange, and prevents the penetration of pathogens (thanks to the smaller pore size). For tissue regeneration (cell penetration and proliferation) membranes are required to be 60–90% porous.^57^ As shown in Figure 4a, the porosity of all membranes produced (change between 73 and 90%) is in the range required for tissue regeneration. Upon examination of all the membranes, the membranes with the highest porosity were the samples with greater gelatin ratios. Besides, it was remarked that the porosities significantly decreased as the bioglass contents of the membranes increased (*p* < 0.05). In the study conducted by Stiglic et al., utilizing citric acid as a cross-linker, it was reported that increasing cross-linking density with increasing cross-linker concentration reduced porosity.^58^ In our study, the citric acid concentration in the blend was kept constant, and the PVA ratio was increased. Upon examination of Figure 2D, it may be asserted that with an elevation in the poly(vinyl alcohol) (PVA) ratio, there is a concurrent augmentation in cross-linking. Therefore, the decrease in the porosity of the membranes may be associated with increasing of PVA ratio (M1 (90.23 ± 1.57%), M5 (88.57 ± 1.86%), and M8 (86.13 ± 2.29%) porosity seen in Figure 4a). In the comparison of the porosity of M11(73.80 ± 2.47%) and M12 (74.45 ± 1.00%) membranes, no significant change was observed (*p* > 0.05). Consequently, it can be concluded that the urea discharged onto the cross-linked membrane through the electrospray method does not have a significant effect on the porosity of the membrane.

### 3.5 Swelling Test

Swelling is an important property for wound dressings and indicates its capacity to absorb exudate, body fluids, and metabolites during the wound healing process.^13,59^ With the increasing of cross-linking density, the interpolymer bonds are strengthened and less porous structures are formed that prevent water molecules from entering the networks.^22^ The results in Figure 4b indicate that there was no significant difference between M1 and M5, but a significant difference was observed between M8 and the M1 and M5 samples. Therefore, as observed in Figure 4b, it can be inferred that an increase in the PVA ratio leads to decreased porosity and, in turn, reduces the degree of swelling of the membranes (e.g., M1, M5, and M8 exhibit swelling percentages of 429.56 ± 11.84, 416.62 ± 35.19, and 343.08 ± 16.57%, respectively). The membranes with different amounts of borosilicate bioglass composition produced by Wu et al., it was observed that while the bioglass content of the membrane was increased, the swelling capacity of the membrane first increased and then decreased.^29^ Similar results were obtained in the study of adding bioglass to chitosan membranes by Sergi et al. It was anticipated to reduce porosity and consequent swelling capacity in wound dressings. However, the analysis results observed that the membrane containing 10% bioglass exhibited greater swelling capacity compared to the membrane containing 5% bioglass. Our study yielded a similar outcome to that observed in the previously mentioned studies. The membrane with 1% and 2% bioglass content (M9 and M10) exhibited the highest swelling capacity, and contrary to expectations, the membrane containing 3% bioglass (M11) showed a statistically significant decrease in the degree of swelling (*p* < 0.05). The swelling degree of the M12 membrane, which is produced by electrospraying urea on M11 membrane, is similar to that of the M11 membrane (*p* > 0.05).

### 3.6 Water Vapor Transmission Rate

**Figure 5:**
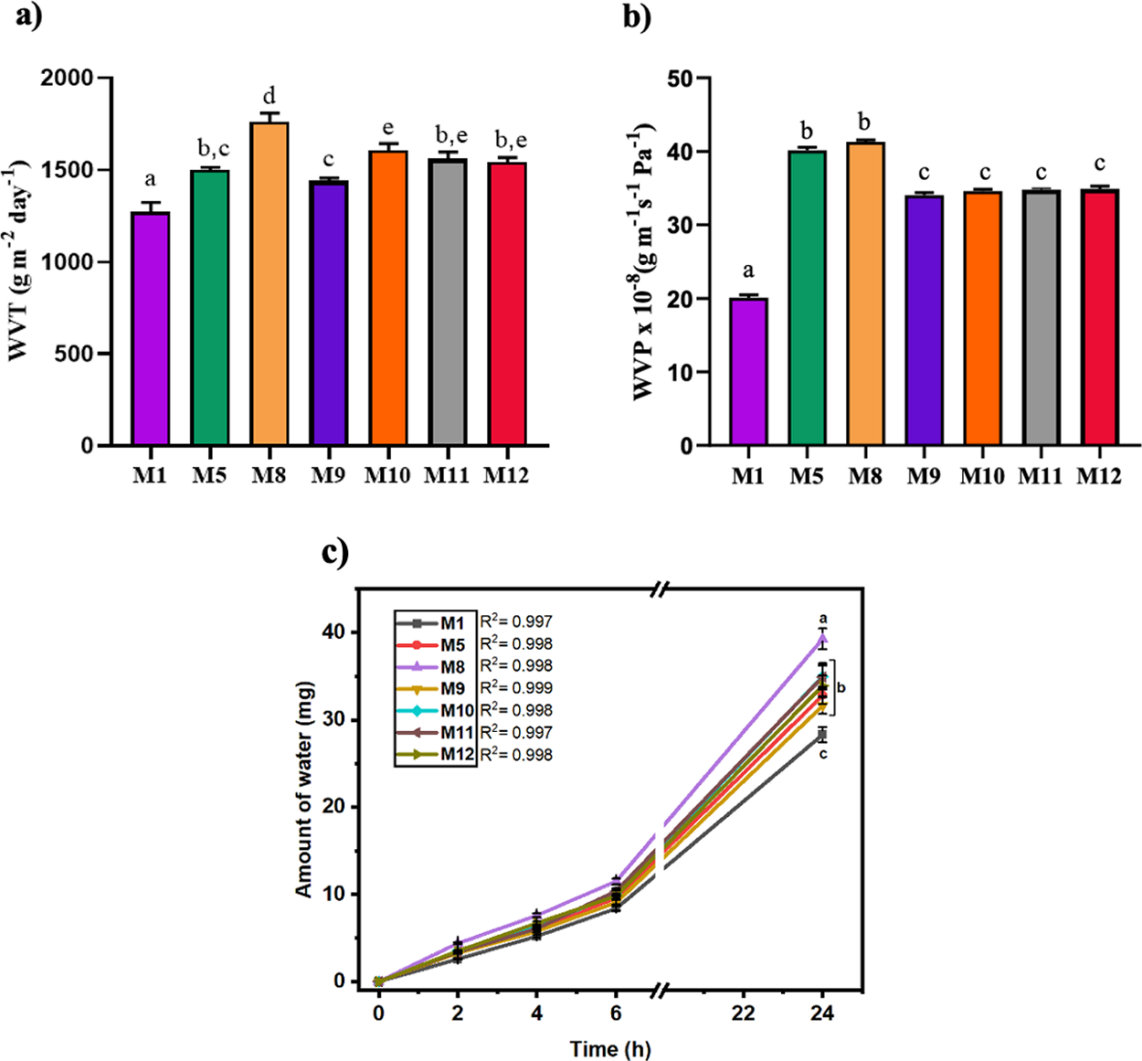
WVT (a), WVT values (b), and water vapor transmission profile (c) of the prepared membranes (data are given as mean ± SEM (*n* = 6)). The different letters on the bar indicate significant differences between values, and the same letters on the bar indicate no difference between values according to Tukey’s multiple comparison test (*p* < 0.05).

WVT and WVP are some of the most important properties and analyses that must be taken into account while developing packaging materials or wound healing biomaterials for use in biomedical applications.^60^ WVT represents the amount of water vapor that migrates from the atmosphere through the membranes within a specified time and is indicative of the membrane’s permeability characteristics.^61,62^ The water vapor transmission profile of the produced membranes, along with the WVT and WVP data, are illustrated in Figure 5.

The WVT of healthy skin is typically around 204 ± 12 g/m^2^ day, whereas in damaged traumatized skin (such as burned skin), this value can be as high as 279 ± 26 g/m^2^ day, while the commercial skin wound dressings developed are in the range of 426–2047 g/m^2^ day.^63^ Upon analysis of the results with respect to the PVA/gelatin ratio in the Figure 5a, it was observed that the membrane with an 80:20 PVA/gelatin ratio exhibited the lowest WVT value (M1), while the highest was observed in the 90:10 ratio (M8) (*p* < 0.05). Among the membranes, those with a 90:10 PVA/gelatin ratio were found to be the most suitable candidates for wound dressing applications. These results can be attributed to the greater WVT value of PVA as compared to gelatin. Hubner et al. reported that the WVT value of hydrogels, which made from PVA and gelatin, did not show a significant change with an increase in the PVA ratio. However, a significant decrease in the WVT value was observed with an increase in the amount of gelatin in the hydrogel.^64^ The reason for the significant decrease in WVT value with increasing gelatin content in the membrane is due to the hygroscopic nature of gelatin, which is sensitive to relative air humidity and can absorb moisture from the environment. In contrast, PVA is less hygroscopic than gelatin, which results in less sensitivity to moisture and hence less impact on the WVT value.^65,66^

The incorporation of BG into the PVA/Gel membranes had a negative impact on WVT, leading to a statically significant decrease in WVT values (M8, M9, M10, and M11 1761.6 ± 42.26, 1440.0 ± 14.04, 1605.8 ± 33.39, and 1563.0 ± 29.88 g/m^2^ day, respectively) (*p* < 0.05). Bioglasses in the membrane can interact with the membranes and create a robust network structure, thus hindering the passage of water molecules through the membranes.^41^ Moreover, the incorporation of nanoparticles into macromolecule structures, such as membranes, can impede the permeability of water vapor as well as permeability of gases by occupying the pores within the structure.^67^ Additionally, the study revealed that the WVT value of the M11 membrane was not statistically significant altered by the addition of urea (M12 membrane, 1541.6 ± 29.88 g/m^2^ day) (*p* > 0.05). It was determined that all the fabricated membranes possessed suitable WVT values for both healthy skin and wound dressings, and the water vapor transmission rate profile was observed to have zero-order kinetics, indicating a constant amount of water vapor permeability per unit time (as depicted Figure 5c).^62^ This result indicates that the transfer of water vapor can remain at a constant rate over time.^68^

WVP is a one of the crucial parameters that is commonly used to assess a membrane’s capacity to limit moisture transfer into the membrane.^61^ The WVP value of a wound dressing should prevent excessive dehydration of the wound because the wound dressing plays an important role in maintaining a proper fluid balance in the wound bed. The WVP serves this by determining the ability of the film to conduct vapor and air through its structure.^64,69^ The addition of bioglass and an increase in the amount of gelatin resulted in a similar trend, resulting in a significant decrease in both WVT and WVP values as depicted Figure 5b (*p* < 0.05). However, the addition of urea did not have a significant effect on either WVP or WVT of membrane (*p* > 0.05).

### 3.7 In Vitro Biocompability Test

**Figure 6:**
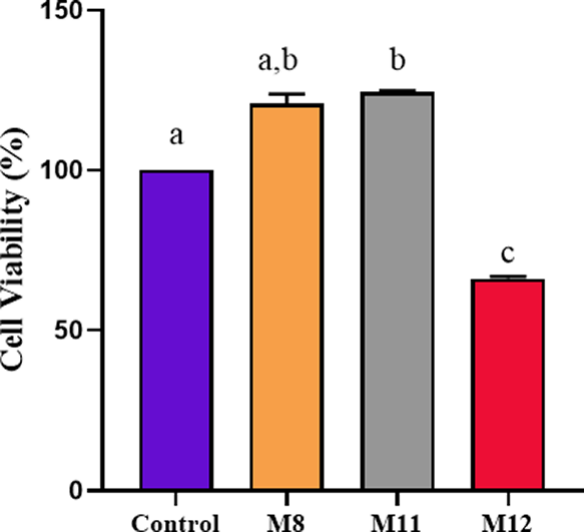
Cell viability (%) results after the fibroblast cells were treated with extracts of electrospun nanofiber membranes (data are given as mean ± SEM (*n* = 6)). The different letters on the bar indicate significant differences between values, and the same letters on the bar indicate no difference between values according to Tukey’s multiple comparison test (*p* < 0.05).

In wound dressing applications, it is important to evaluate the cytotoxic effects of nanofiber membranes on fibroblast cells. In order to evaluate the biocompatibility of nanofibrous membranes, the MTT assay was performed using L929 mouse fibroblast cell lines. Cell viability results after incubation with membrane extracts for 24 h are given in Figure 6. The cell viability in the wells with membrane extracts and control groups was calculated using eq 5. The cell viability of the extracts of M8, M11, and M12 nanofibers was 120.83 ± 4.25, 124.48 ± 0.47, and 66.21 ± 0.91%, respectively, while the cell viability of the control group was 100%. According to ISO 10993–5:2009 standards, substances are considered noncytotoxic if cell viability is greater than 70%. It was observed that M8 and M11 nanofiber membranes do not show any cytotoxic effect on cells. These results indicated that the PVA and gelatin in the membranes showed biocompatible behavior. Further, M11 membrane increased proliferation of fibroblast cells owing to including BG which is known to contribute to the acceleration of fibroblast cell proliferation.^70,71^ The cytotoxicity result of papain-urea-PVA electrospun nanofibers prepared by Shoba et al. was found to be approximately 80% in HaCaT cells.^72^ Ghorai et al. found the cell viability of polyurethane-urea based electrospun nanofiber membranes to be 90% and above.^73^ On the other hand, Krysiak et al. reported that polymer fibers with urea generated by electrospinning and electrospraying techniques exhibited a toxic effect on keratogenic cells in vitro. They stated that the number of keratinocytes started to decrease when the concentration of urea reached its maximum. They also concluded that the direct contact of the cells with the urea caused the cytotoxic effect.^37^ The cytotoxicity of mild polyurethane-urea foam was previously tested on NIH 3T3 cells by Liu et al., and the cell viability was found to be 68.64% (24 h). They stated that mild polyurethane-urea foam had a slightly cytotoxic effect on cells.^74^ The M12 membrane related to the presence of urea showed a slight cytotoxic effect on the L929 mouse fibroblast cells. It was thought that the slightly toxic effect of the M12 membrane may be based on the direct contact of urea with cells due to the usage of free-form urea on the fiber. As a result of in vivo study (the results are displayed in Figure 10), it was observed that M12 reduced inflammation and increased collagen deposition in the wound site.

### 3.8 In Vivo Evaluation of Developed Wound Dressings

#### 3.8.1 Clinical Follow-Up and Wound Area Results

**Figure 7:**
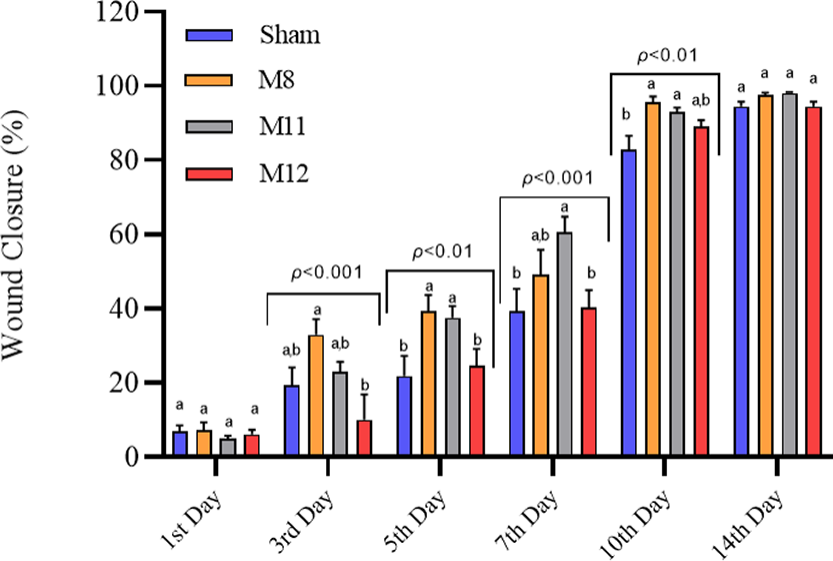
Wound closure rates of Sham, M8, M11 and M12 groups at different time points (days 1, 3, 5, 7, 10, and 14). Data are given as mean ± SEM (*n* = 12 for 1, 3, 5, 7, 10 days, *n* = 6 for 10 and 14 days). The different letters on the bar indicate significant differences between values, and the same letters on the bar indicate no difference between values according to Tukey’s multiple comparison test for each day (*p* < 0.01 for the 5th and 7th days, *p* < 0.001 for the 3rd and 10th days, no significant differences on the 1st and 14th days).

**Figure 8:**
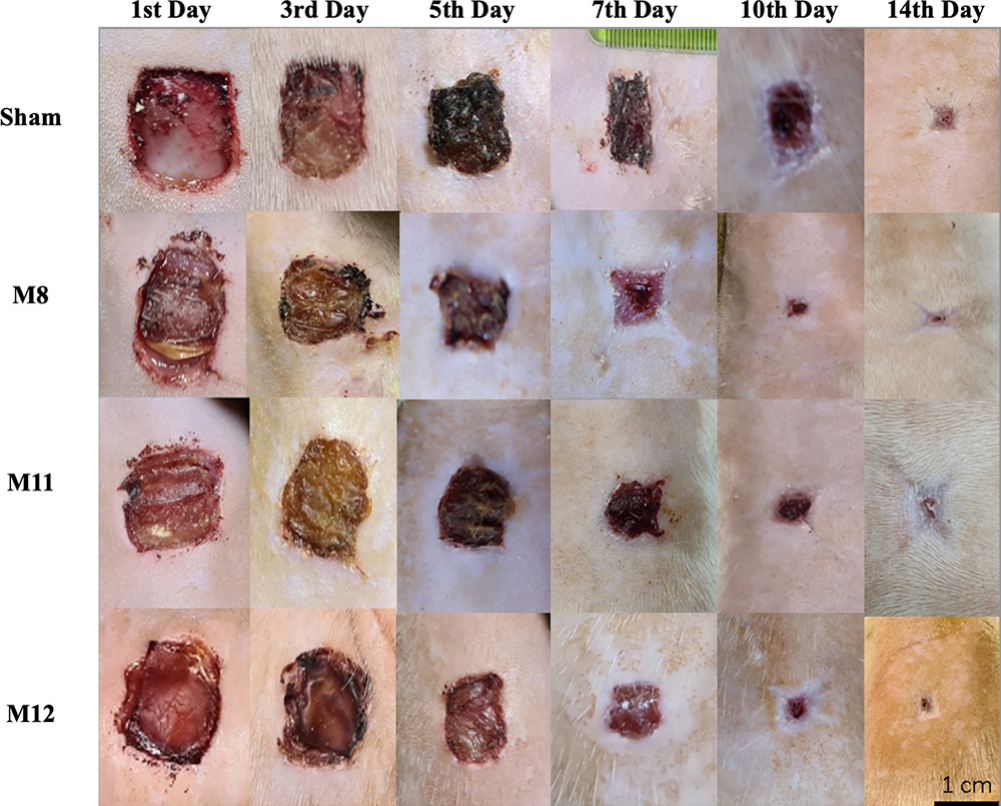
Wound images of Sham, M8, M11, and M12 groups at different time points (days 1, 3, 5, 7, 10, and 14).

Measurements of the wound area are included in studies as the most radical way to assess wound healing clinically.^75−77^ The study employed the Wistar rat full-thickness excisional wound model to assess the potential for wound healing of three distinct membranes (M8, M11, and M12) utilized as wound dressings. The time-dependent wound closure rate results and the appearance of the wound areas are presented in Figures 7 and 8, respectively. When the wound healing rates were analyzed according to the days (Figure 7), the wound closure percentage of the M8 membrane-treated group was observed to be the highest on the third day. However, no significant difference was observed compared to that in sham. Moreover, on the same day, the M12 and M11 groups were similar to the sham group (*p* > 0.001). In the following days, it was determined that the wound healing rate of M11 membrane was increased. On day 7 postsurgery, it was revealed that the wound closure rate of the M11 treated group (61%) was significantly higher than that of the sham group (39%) (*p* < 0.001); on the other hand, there was no statistically significant difference between the sham and M8 (49%) or M12 (40%) treated groups. This can be explained by the fact that BG nanoparticles in the biodegraded membrane interact with physiological body fluids over time. On day 10 postsurgery, the M8 (95%) and the M11 (93%) groups showed a significantly higher (*p* < 0.01) wound closure rate than the sham (82%); however, the M12 group (90%) did not. There was no statistically significant difference in the wound closure rates between the M8, M11, and M12 groups. Thus, it may be concluded that wound healing accelerated for these groups based on the wound closure rates at the 10th day. The effect of the M8 membrane on wound healing can be explained by its ability to mimic the ECM found in nature and contains a hemostatic agent (gelatin) which facilitates fibroblast migration to the wound site and encourages cell proliferation by fostering an inflammatory phase.^78^ On the other hand, the M11 membrane contributes to promoting wound healing by containing 45S5 BG, in addition to having the same characteristics as the M8 membrane. According to a study by Yu et al., Si, Ca, and P ions released from bioglasses into physiological body fluids stimulate the secretion of growth factors (bFGF, VEGF, and EGF) to accelerate angiogenesis and fibroblast migration. They also increase the synthesis of collagen I and fibronectin. Thus, it has been claimed that bioglasses play a significant role in accelerating the healing of wounds.^71^ In another study, Sharaf et al. proposed using bioglass-loaded cellulose acetate electrospun nanofiber membrane to accelerate diabetic rats’ wound healing because of the beneficial effects of bioglass.^79^ It was predicted that the M12 group, like the M11 membrane, would accelerate wound closure, because it contains BG and PVA/gelatin. However, related to the fact that it contains urea, it has been noticed that the healing rate is slightly lower than that of the M11 group. This is because urea regulates the proliferation of epidermal cells by reducing DNA synthesis in basal cells and prolonging the formation time of epidermal cells after mitosis.^80,81^ On day 14 postsurgery, the wound closure rates in the Sham, M8, M11, and M12 groups were 94, 97, 98, and 94%, respectively; it was determined that the wound was almost closed in all groups, and there was no significant difference between the groups. Images of the wound sites in Figure 8 demonstrate that the membranes have the ability to absorb exudate and are biodegradable. Maintaining moisture in the wound bed is one of the fundamental functions that wound dressings need to provide. A wound that is exposed to air directly causes it to dehydrate and develop a scab. Research has indicated that healing happens more quickly in a humid environment.^82^ It was observed that the M12 membrane reduced the formation of scabs due to the urea that it contains (shown in Figure 8). Thus, it may have provided a more moist wound environment than the others. Choi et al. previously observed that the use of polyurethane-urea based liquid bandage material in wound healing maintained the moisture of the wound area at an adequate level, and accordingly, the formation of wound scabs was prevented.^82^ It is also well-known that the absence of a scab reduces scarring.^83^ Urea is a natural moisturizing factor (NMF) found in the skin’s outermost layer, and urea is known to act as a moisturizer at lower concentrations, such as 1–20%.^84^ Additionally, urea is used to regulate the barrier function of human skin by controlling the mRNA expression of certain genes related to keratinocyte differentiation, and it contributes to strengthening the skin’s immune system by increasing the synthesis of antimicrobial peptides in the epidermis. Even in normal skin, the use of urea helps these peptides improve barrier function and strengthen the skin’s immune system.^84^ In accordance with the above-mentioned features of urea, when the wound images (Figure 8) are examined more carefully, it has been observed that the regenerated skin after M12 membrane treatment is more similar to normal skin without an uneven scar compared to other membranes. Besides, the analysis of inflammation in Figure 10A and collagen deposition shown in Figure 11B supports this observation. As a result, it can be suggested that M12 membrane can be used to reduce scar formation because it may have an inhibitory effect on the development of normal, hypertrophic and keloid scars with the synergistic effect of BG and urea.

#### 3.8.2 Histopathological Analysis

**Figure 9:**
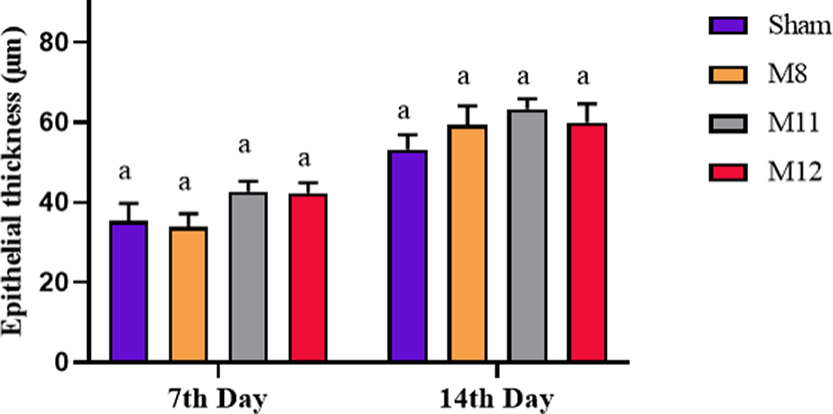
Epithelial thickness of Sham, M8, M11, and M12 groups at different time points (days 7 and 14). Data are given as mean ± SEM (*n* = 6). The same letters on the bar indicate no difference between values according to Tukey’s multiple comparison test for each day (*p* > 0.05).

Histopathological analysis was carried out on days 7 and 14 following transplantation in order to examine wound healing more intently. Sections from the wound area were stained with HE to measure epithelial thickness for investigating re-epithelialization; the results are shown in Figure 9. On the seventh day, the mean epithelial thicknesses of the Sham, M8, M11, and M12 groups were measured and determined to be 35.4 ± 4.32, 33.9 ± 3.27, 42.7 ± 2.61, and 42.4 ± 2.49 μm, respectively. On the 14th day, the groups’ epithelial thicknesses were measured to be 53.2 ± 3.68, 59.3 ± 4.84, 63.3 ± 2.55, and 60.0 ± 4.61 μm, respectively. M11 and M12 groups had higher epithelial thicknesses on both days. However, there was no significant difference between the groups on both days. Furthermore, despite the fact that the wound size was the same in all groups on the 14th day macroscopically, according to the microscopic analysis observation, the eroded area continued in all of the sham groups and some of the M8 groups, and full-thickness epithelialization was observed in the entire M11 groups and almost all of the M12 groups.

**Figure 10:**
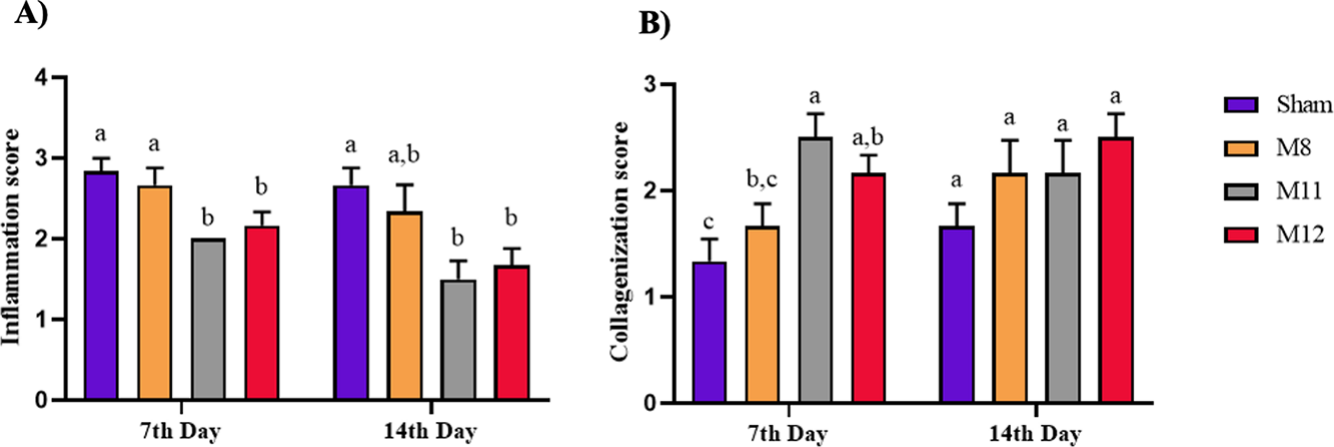
(A) Inflammation and (B) collagenization levels of Sham, M8, M11, and M12 groups at different time points (days 7 and 14). Data are given as mean ± SEM (*n* = 6). The different letters on the bar indicate significant differences between values, and the same letters on the bar indicate no difference between values for each day according to the Kruskal–Wallis test followed by Dunn’s test for multiple comparisons (*p* < 0.05 for inflammation scores on the 7th and 14th days, *p* < 0.01 for collagenization scores on the 7th day, and no significant differences in collagenization scores on the 14th day *p* > 0.05).

**Figure 11:**
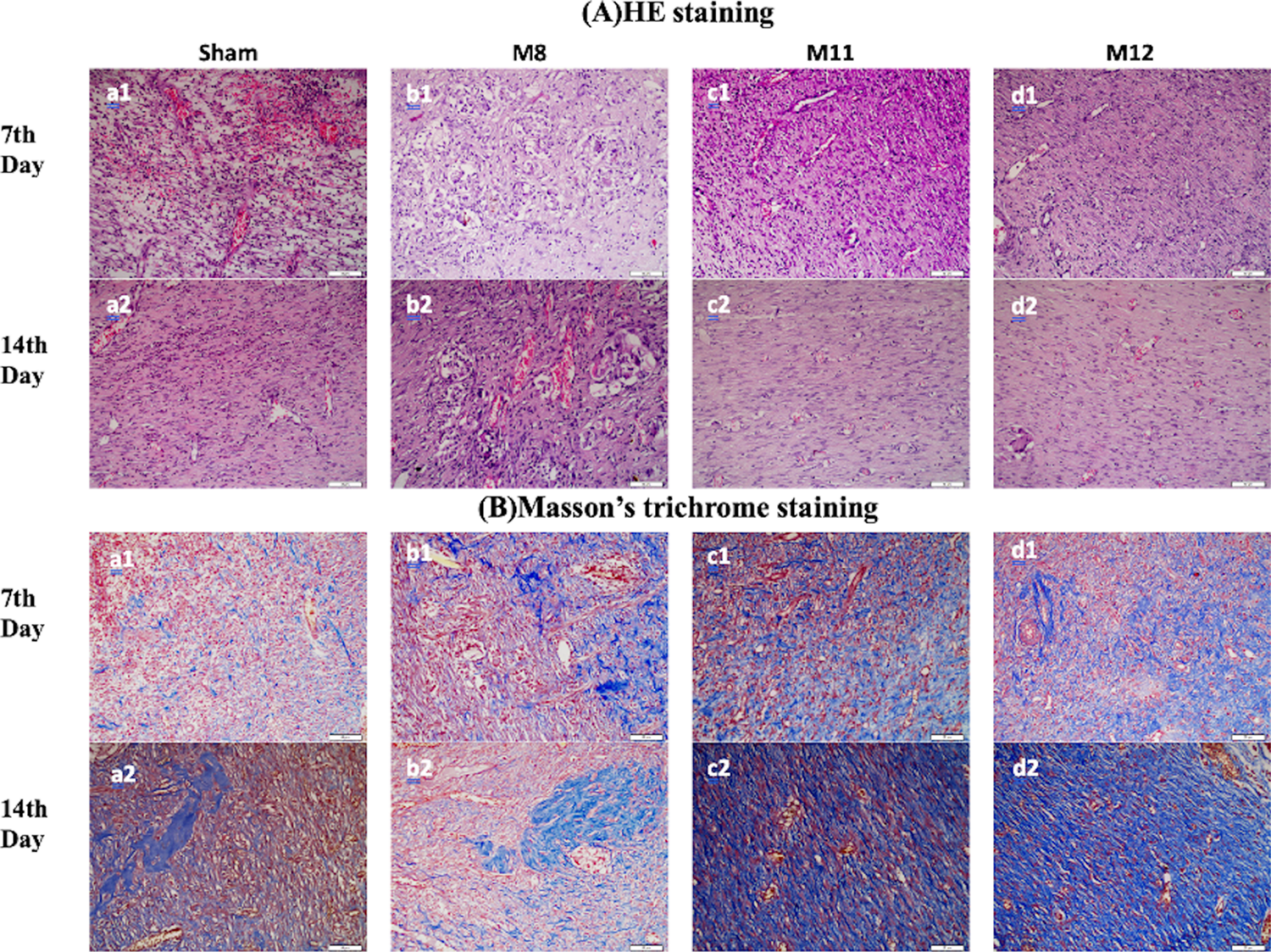
(A) Hematoxylin-eosin (HE) staining images of wound sites and (B) Masson’s trichrome (MT) staining images of the wound sites.

The inflammatory phase is an essential stage in the healing of wounds because of required to prevent pathogens and remove dead tissue.^85^ As inflammation decreases in the wound area, new blood vessels and connective tissue begin to form. Consequently, the wound area narrows, and the proliferation phase begins.^86^ Prolonged inflammation can affect the normal progression of wound healing, resulting in a delay in the healing process and leading to abnormalities in the activation and differentiation of keratinocytes.^85^ Additionally, inflammation plays a role in modulating collagen synthesis, and the intensity of inflammation has been associated with an increase in the final scar of the wound.^86^ Images of wound sites stained with hematoxylin-eosin (HE) in order to assess the inflammation are presented in Figure 11A and the results are given in Figure 10A. It was concluded that the inflammatory reaction was significantly suppressed in the groups treated with M11 and M12 membranes compared to the Sham group (*p* < 0.05). On day 14, it was observed that moderate to severe inflammation persisted in both the M8 and Sham groups, the Sham group predominantly exhibited acute inflammation. Neutrophils must be replaced by lymphocytes and plasma cells on 3–fourth day of the normal wound healing process. The presence of neutrophil leukocytes after these days is an indicator of acute inflammation and is one of the factors that delay wound healing.^87^ Examining the inflammatory cells in the M11 and M12 groups, we found that lymphocytes and plasma cells were the most prevalent types of inflammatory cells. Accordingly, neither the M11 nor the M12 groups showed prolonged acute inflammation. It is known that the ionic dissolution products of 45S5 bioglass influence increasing the expression of anti-inflammatory factors in macrophages and thus speeding up the wound healing process by reducing the inflammatory response.^88^ The results suggest that M11 and M12 membranes expedite wound healing by reducing the inflammatory response in the presence of 45S5 bioglass.

The most prevalent protein in connective tissue and one that is produced by fibroblasts, collagen, is crucial for tissue regeneration because it helps the repaired skin regain its mechanical strength, elasticity, and functionality. Furthermore, it is necessary to maintain the dynamic balance between the synthesis and degradation of collagen to prevent scar formation.^89^ To assess the collagen deposition that takes place 7 and 14 days after transplantation, images of the wound areas stained with Masson’s trichrome (MT) are provided in Figure 11B, and results are given in Figure 10B. On the seventh day, the groups’ collagen accumulation was examined, and the M11 and M12 groups showed significantly increased collagen formation (*p* < 0.01) than the sham group. This can be explained by the fact that enhanced fibroblast proliferation triggered by Si, Ca, and P ions released from BG has a noticeable impact on collagen synthesis.^71^ Although the collagen depositions of all groups were similar at the end of the 14th day (*p* > 0.05), collagen bundles were observed to distribute more regularly in the M11 and M12 groups compared to Sham and M8 groups as Figure 11B clearly shows. This suggests that hypertrophic scars or keloid tissue may develop in the sham and M8 groups in the future. Myofibroblasts undergo apoptosis after re-epithelialization in wound healing to reduce excessive collagen deposition for preventing excessive scar formation, such as keloids and hypertrophic scars. It is well-known that BG encourages the formation of regular, properly positioned collagen fibers. Besides, BG plays an effective role in reducing excessive scar formation such as hypertrophic scars and keloids by inhibiting the differentiation from fibroblast to myofibroblast.^88^ Nevertheless, at concentrations of 6–30%, urea exhibits proteolytic activity.^81^ It is probable that the proteolytic activity of urea inhibited the development of unequal and coarse collagen. Furthermore, the results of the cytotoxicity analysis showed that the M12 membrane had a slightly toxic effect on fibroblast cells. In contrast, the inflammation and collagenization results showed clearly that the incorporation of urea in the M12 membrane may be tolerated in vivo. Overall, these results show that PVA/Gel/BG/Urea membranes exhibited biocompatible properties although it was with a slight cytotoxic effect.

##### 3.8.2.1 Immunohistochemical Staining

**Figure 12:**
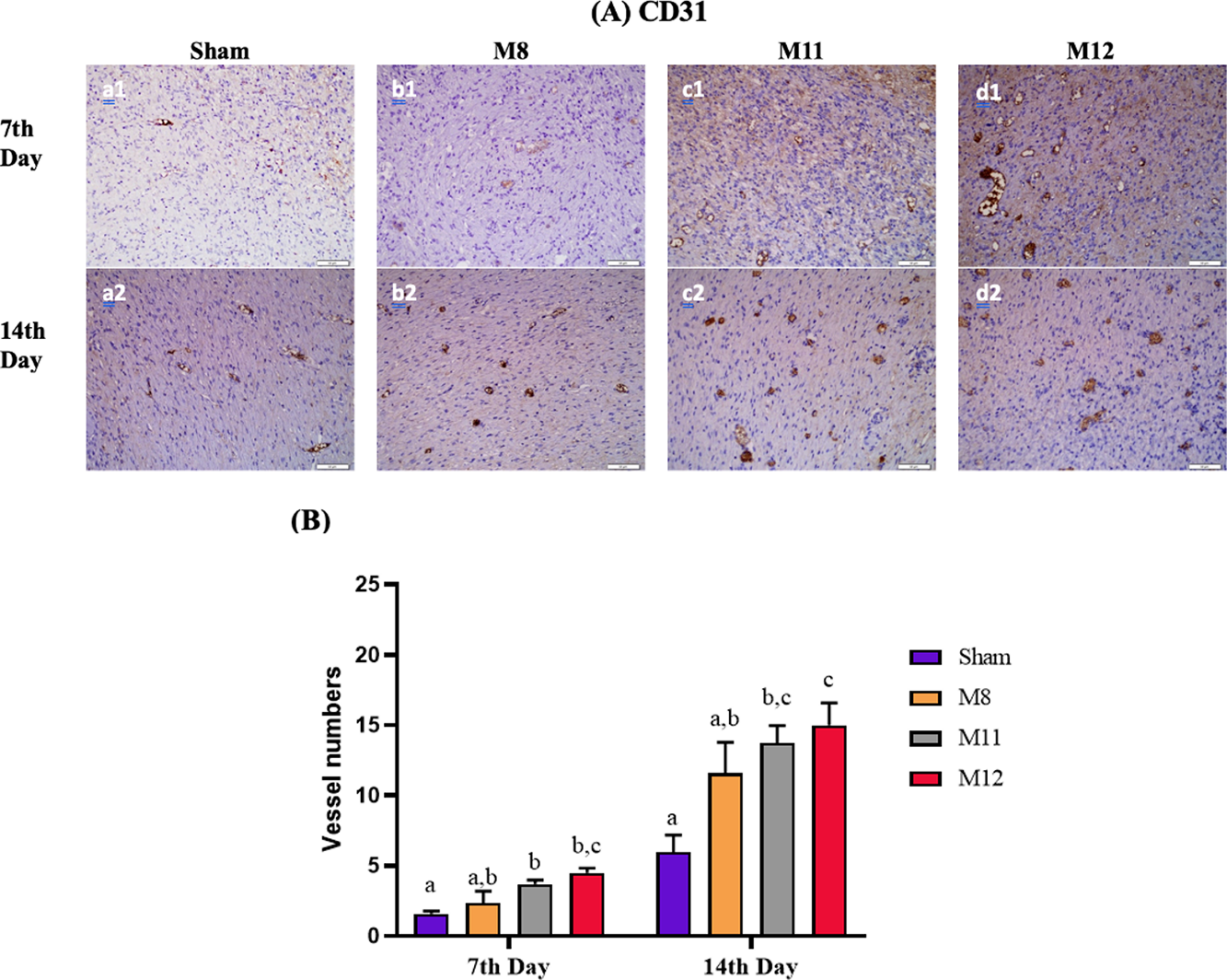
(A) CD31 immunohistochemical staining of wound sites at different time points (days 7 and 14). (B) Number of newly formed vessels of Sham, M8, M11, and M12 groups at different time points (days 7 and 14). Data are given as mean ± SEM (*n* = 6). The different letters on the bar indicate significant differences between values, and the same letters on the bar indicate no difference between values according to Tukey’s multiple comparison test (*p* < 0.05).

Granulation tissue, which contains many microvessels, is formed during the proliferative phase of wound healing. Angiogenesis contributes to the wound repair process by providing oxygenation and nutritional support, the rapid arrival of reparative cells to the wound area, and the balanced elimination of the formed residues.^90^ To evaluate the vascularization of wounds treated with membranes during the healing process, CD31 staining was conducted to reveal the newly formed blood vessels (shown in Figure 12A,B. The vessel amounts in the M11 and M12 groups were found to be significantly higher than in the sham group in both days (*p* < 0.05). Additionally, there was no significant difference observed between M11 and M12. As a result, the M11 and M12 membranes increased vascularization owing to the inclusion of BG, which promotes angiogenesis.^1,91,92^ Urea concentration was found to increase the size and rate of granulation tissue formation while delaying the rate of epithelialization according to research on the effect of urea on wound healing conducted by Olson et al.^93^ Based on our findings, the M12 membrane exhibited the highest level of vascularization on both days. It can be concluded that using bioglass in conjunction with urea can partially enhance vascularization in comparison with using bioglass alone.

## 4 Conclusions

The notable attributes of bioglass, particularly in the realm of soft tissue regeneration, have garnered significant attention in contemporary scientific inquiry. Furthermore, urea has enjoyed sustained preference over the years owing to its substantive contributions to the field of wound healing. In the context of this study, multifunctional and biomimetic nanofibrous membranes were successfully fabricated with the integration of bioglass and urea components via green electrospinning. Findings from *in vivo* and histopathological analysis reveal that functionalization of the PVA/Gel membrane with bioglass contributed to accelerating wound healing with its anti-inflammatory, increased collagenization, and neovascularization features. Additionally, the incorporation of urea was observed to mitigate scab formation, suggesting its potential to attenuate uneven scarring. Overall, PVA/Gel/BG and PVA/Gel/BG/Urea membranes exhibited accelerated wound healing with their biodegradable, biocompatible features, and the ability to absorb the exudate. Therefore, they may be regarded as good candidates for wound healing applications. Furthermore, due to their promising features, the PVA/Gel/BG/Urea membrane may be recommended as a wound dressing for antiscar treatment applications. This is especially important considering the aesthetic concerns that exist today.

## Supporting Information Available

Scanning electron microscopy (SEM) images of membranes at ×30k magnification, fiber diameter distribution histogram graphics of membranes, table of the average elemental composition of M9, M11, and M12 membranes, and energy-dispersive X-ray spectroscopy (EDS) spectra of the M9, M11, and M12 membranes (PDF)

## Abbreviations

**M1**: (PVA:Gel (80:20) - CA (15%))
**M2**: (PVA:Gel (80:20) - CA (10%) - BG (1%))
**M3**: (PVA:Gel (80:20) - CA (15%) - BG (1%))
**M4**: (PVA:Gel (80:20) - CA (20%) - BG (1%))
**M5**: (PVA:Gel (85:15)- CA (15%))
**M6**: (PVA:Gel (85:15)- CA (15%) - BG (1%))
**M7**: (PVA:Gel (85:15)- CA (15%) - BG (2%))
**M8**: (PVA:Gel (90:10)- CA (15%))
**M9**: (PVA:Gel (90:10)- CA (15%) - BG (1%))
**M10**: (PVA:Gel (90:10)- CA (15%) - BG (2%))
**M11**: (PVA:Gel (90:10)- CA (15%) - BG (3%))
**M12**: (PVA:Gel (90:10) CA (15%) - BG (3%) - Urea (10%))
