# Supplementary material for: Green Electrospun Poly(vinyl alcohol)/Gelatin-Based Nanofibrous Membrane by Incorporating 45S5 Bioglass Nanoparticles and Urea for Wound Dressing Applications: Characterization and In Vitro and In Vivo Evaluations
Source: ACS Omega. 2024 May 2;9(19):21187–203. doi: 10.1021/acsomega.4c01102 (PMC11097359; doi:10.1021/acsomega.4c01102)
Supplement: Supplementary file 1 — ao4c01102_si_001.pdf [file ao4c01102_si_001.pdf]

Green Electrospun Poli(vinyl alcohol)/ Gelatin Based Nanofibrous Membrane by Incorporating 45S5 Bioglass nanoparticle and Urea for Wound Dressing Applications: Characterization , *In Vitro* and *In Vivo* Evaluations

Tülay Merve TEMEL-SOYLU[1*], Ceren KEÇECILER-EMIR[1,2,], Taha RABABAH[3], Cem ÖZEL[1], Sevil YÜCEL[1], Yeliz BASARAN-ELALMIS[1], Dilan ALTAN[1], Ömer KIRGIZ[4], İlke Evrim SEÇİNTİ[5], Ufuk KAYA[6], Muhammed Enes ALTUĞ[4]

[1]Faculty of Chemical and Metallurgical Engineering, Department of Bioengineering, Yildiz Technical University, İstanbul, Turkey
[2]Faculty of Rafet Kayis Engineering, Genetic and Bioengineering Department, Alanya Alaaddin Keykubat University, Antalya, Turkey
[3]Nutrition and Food Technology Department, Jordan University of Science and Technology, Irbid, Jordan
[4]Faculty Of Veterinary, Department Of Clinical Sciences, Hatay Mustafa Kemal University, Hatay, Turkey
[5]Faculty of Medicine, Department of Pathology, Hatay Mustafa Kemal University, Hatay, Turkey
[6]Faculty Of Veterinary, Department Of Biostatistics, Hatay Mustafa Kemal University, Hatay, Turkey

***Correspondence:** Tülay Merve TEMEL-SOYLU, Faculty of Chemical and Metallurgical Engineering, Department of Bioengineering, Yildiz Technical University, İstanbul 34220, Turkey. Phone: +90 0(531) 932 18 93. Email: t.mervesoylu@gmail.com (Orcid ID: 0009-0006-5374-9316)

Scanning Electron Microscopy (SEM)

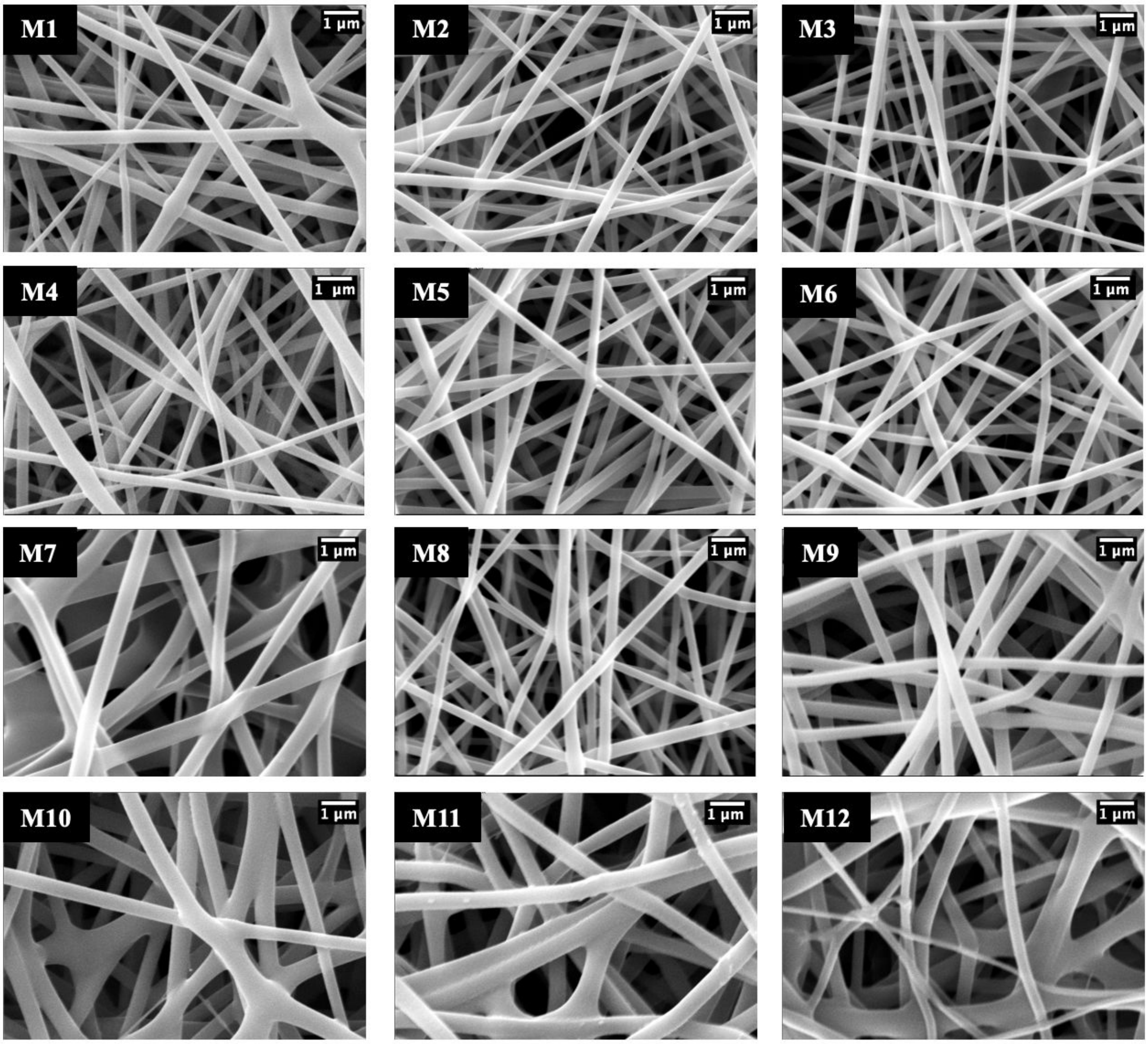

Figure S1. SEM images of PVA- Gelatin membranes with different volumes and contents at x30k magnification.

Fiber Diamater of Membranes

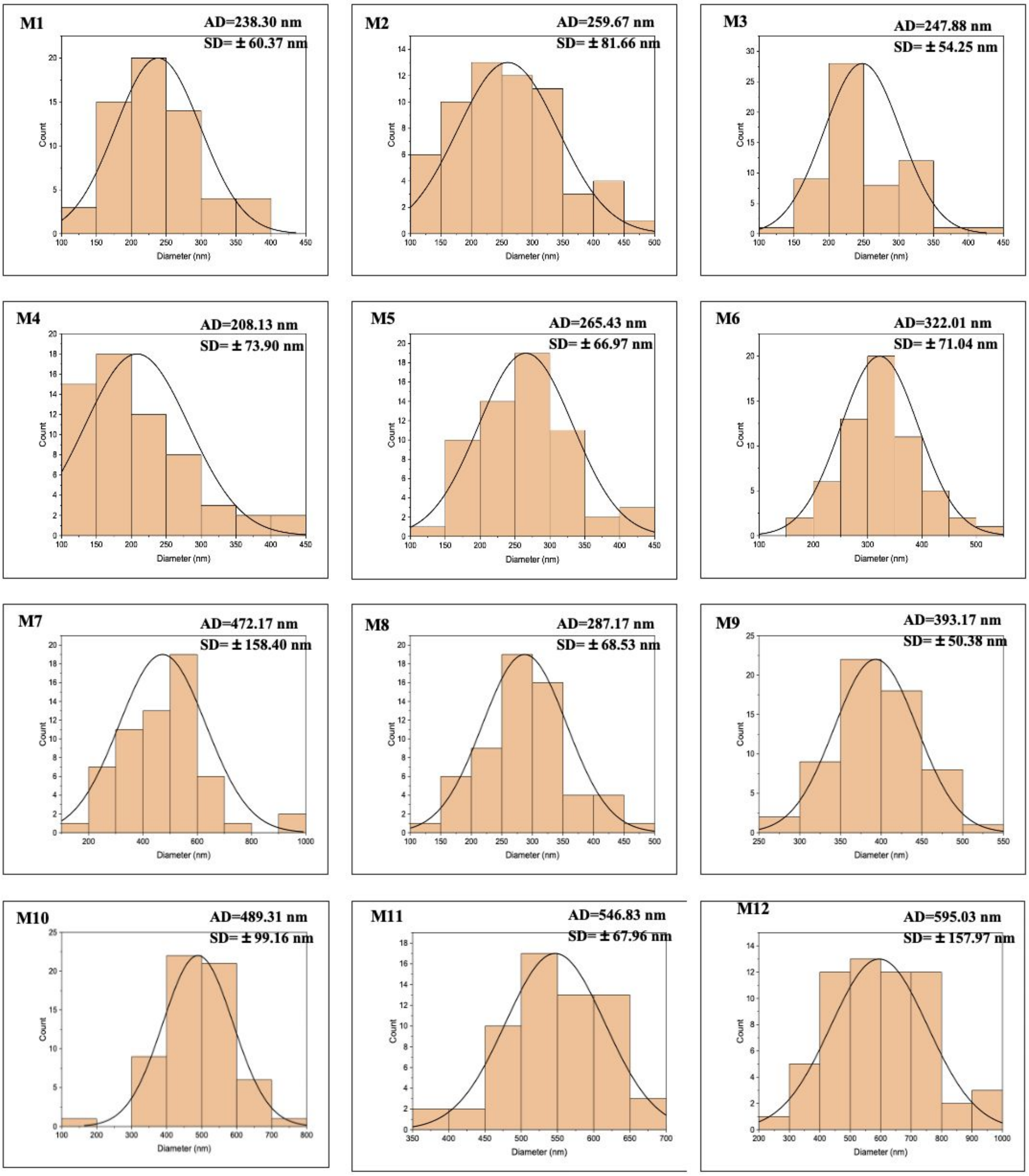

Figure S2. Fiber diameter distribution histogram graphics of produced nanofiber membranes

Energy Dispersive Spectrometry (EDS)

Table S1. The average elemental composition of M9, M11 and M12 Membranes

Sample	45S5 Bioglass Content(%)	Urea Content(%)	Element (wt%)				
			Sodium	Silisyum	Oxygen	Calcium	Nitrogen
M9	1	-	1,76	1,61	14,77	1,38	0
M11	3	-	2,60	1,98	18,11	2,36	0,46
M12	3	10	7,70	0,31	34,91	1,06	7,70

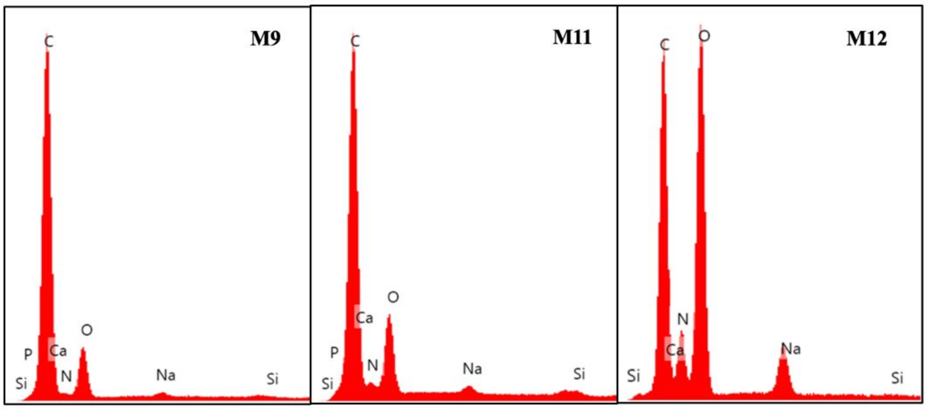

Figure S3. EDS spectrum of M9, M11 and M12 Membranes
